# Computed tomography imaging of a leopard tortoise (*Geochelone pardalis pardalis*) with confirmed pulmonary fibrosis: a case report

**DOI:** 10.1186/1751-0147-55-35

**Published:** 2013-04-23

**Authors:** Chee Kin Lim, Robert M Kirberger, Emily P Lane, Dorianne L Elliott

**Affiliations:** 1 Diagnostic Imaging Section, Department of Companion Animal Clinical Studies, Faculty of Veterinary Science, University of Pretoria, Private Bag X04, Onderstepoort, 0110, South Africa; 2 Research and Specialised Services, National Zoological Gardens of South Africa, P.O. Box 754, Pretoria, 0001, South Africa; 3 Bird and Exotic Animal Hospital, Onderstepoort Veterinary Academic Hospital, University of Pretoria, Private Bag X04, Onderstepoort, 0110, South Africa

**Keywords:** Chelonian, Pulmonary fibrosis, Radiography, Computed tomography

## Abstract

An approximately 20-year-old, female Leopard tortoise *(Geochelone pardalis pardalis)* was presented with dypsnea, wheezing, anorexia and depression. Whole body radiographs revealed generalized diffuse unstructured ‘interstitial lung pattern’ with thickened pulmonary septae while computed tomography (CT) showed emphysematous lung parenchyma and thickened pulmonary septae bordered by irregular ground-glass opacity with smaller areas of ‘honeycombing’. These imaging findings together with histopathologic findings were compatible with chronic, extensive ‘interstitial’ pulmonary fibrosis.

## Background

Pulmonary fibrosis is a chronic and progressive interstitial lung disease with a poor prognosis [1-3] and can be the end-stage of various pulmonary conditions. Although this condition has been reported in humans [4], dogs [5,6] and cats [7,8], its pathophysiology is poorly understood and its exact etiology is unknown [1-7]. In veterinary medicine, the diagnosis of pulmonary fibrosis is made based on clinical findings, diagnostic imaging and exclusion of other cardiorespiratory illnesses. Definitive diagnosis requires histopathologic examination [1,6]. Recently, diagnostic biomarkers such as endothelin-1 and procollagen type III amino terminal propeptide have been shown to be elevated in pulmonary fibrosis [2,9] but further studies are necessary to consolidate the approval of use of such biomarkers for the diagnosis of pulmonary fibrosis.

The radiographic and computed tomographic features of pulmonary fibrosis have been well described in dogs [5,6,10] and humans [11]. High-resolution CT is considered crucial for diagnosis of pulmonary fibrosis in humans [11] and dogs [10].

Although CT of chelonians has been found feasible [12-14] and normal computed tomographic anatomy of the respiratory system of the Egyptian tortoise (*Testudo kleinmanni*) [15] as well as loggerhead sea turtle (*Caretta caretta*) [16] has been reported, there is no computed tomographic description of pulmonary fibrosis in chelonians.

This report describes the radiographic and computed tomographic appearance of histologically confirmed pulmonary fibrosis in a chelonian.

## Case presentation

An approximately 20-year-old, 5.6 kg, female Leopard tortoise *(Geochelone pardalis pardalis)* was presented for anorexia and depression. The patient was housed in a suburban garden and had access to a variety of garden plants as well as cut fruit, vegetables and small amount of soaked dog kibbles. On clinical examination, she was dyspneic and wheezing with each breath. There was no oronasal discharge and the oral cavity was within normal limits. Her carapace was slightly deformed with tenting of the scutes suggesting long term malnutrition with an excess of protein in diet.

## Diagnostic imaging

**Figure 1:**
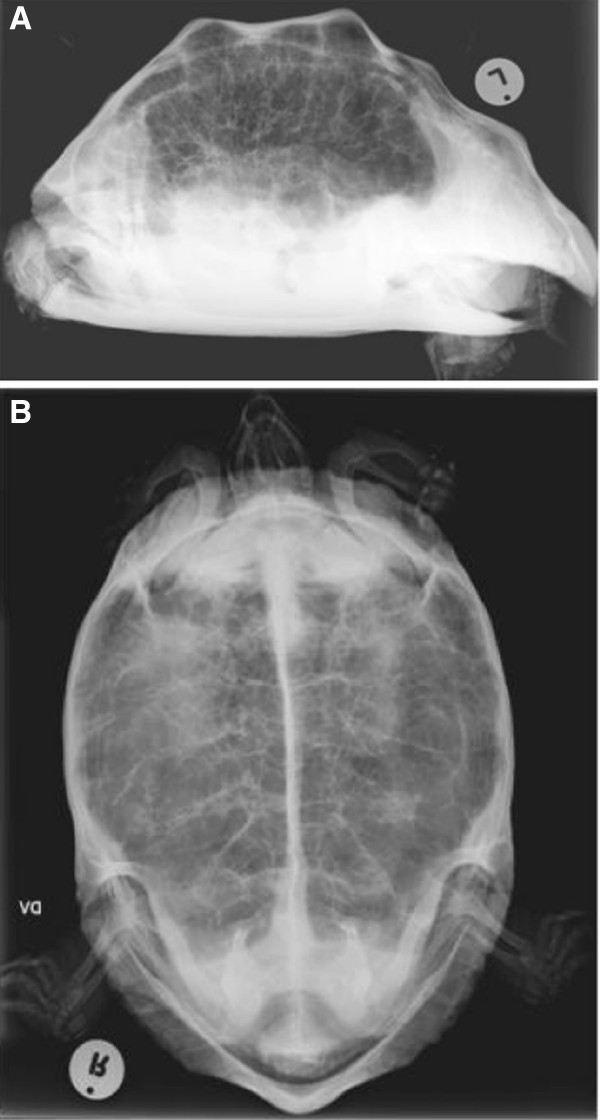
**Whole body radiographs of the affected Leopard tortoise with suspected pulmonary fibrosis.** (**A**) Lateral horizontal beam and (**B**) dorsoventral radiographs of a Leopard tortoise. Note the diffuse unstructured ‘interstitial lung pattern’ in both radiographs.

Whole body radiographs revealed a generalized diffuse unstructured ‘interstitial lung pattern’ with thickened pulmonary septae (Figure 1A and B) and a diagnosis of suspected pulmonary fibrosis was made.

Whole body helical CT (Siemens Emotion Duo, Siemens Medical Systems, Forchheim, Germany) was performed with the patient in ventral recumbency and strapped to the CT table using Velcro strips without any chemical restraint. A piece of alcohol-soaked cotton wool was placed cranial to the epiplastron to prevent the head from protruding and to limit movement. Contiguous, transverse 3 mm thick slices were acquired (mAs: 28; kVp: 130; matrix: 512 × 512; pitch 1.9; rotation time 0.8 sec) using lung (window level (WL) -600; window width (WW) 1200) and mediastinum (WL 40, WW 400) algorithms. Dorsal and sagittal multiplanar reformatted (MPR) images were reconstructed and compared to radiographic findings.

**Figure 2:**
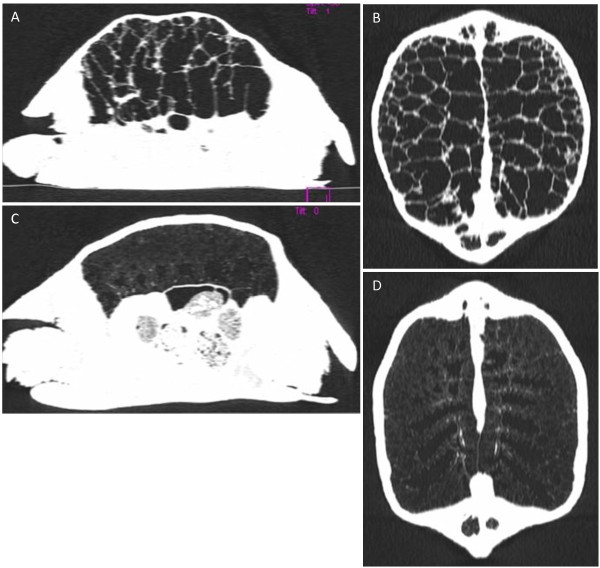
**Comparison of sagittal and dorsal MPR CT images of the affected Leopard tortoise with the normal Leopard tortoise in lung window (WL −600, WW 1200).** The affected tortoise (**A** &**B**) shows the thickened pulmonary septae and the normal tortoise (**C** &**D**) shows a normal diffuse reticular lung pattern.

**Figure 3:**
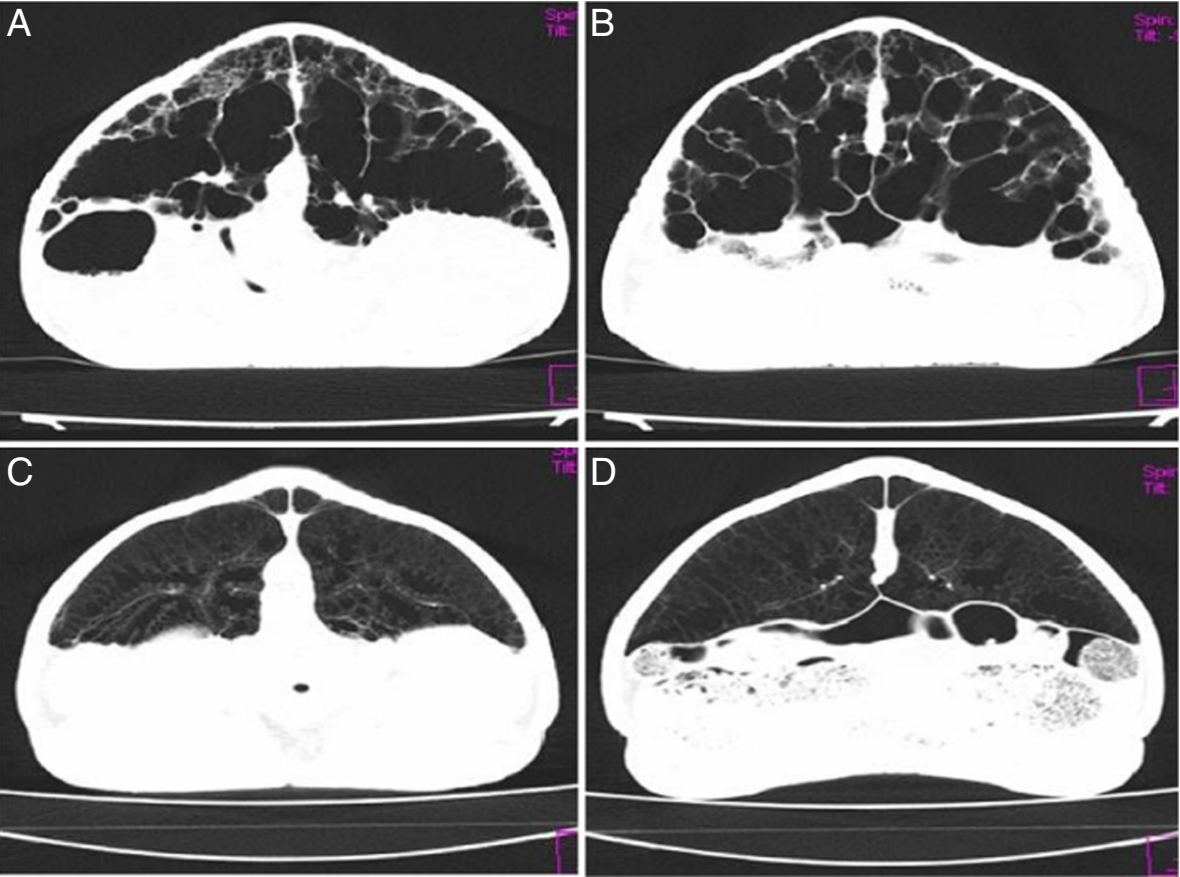
**Comparison of transverse CT images of the affected Leopard tortoise with the normal tortoise in lung window (WL -600, WW 1200).** Note the emphysematous lung with patchy 'ground-glass' attenuation bordering the thickened pulmonary septae with smaller areas of 'honeycombing' in the affected tortoise (**A** &**B**). Normal diffuse reticular lung pattern in normal tortoise (**C** &**D**).

The lungs appeared emphysematous with irregularly thickened pulmonary septae (Hounsfield Unit (HU) -114 to 389) (Figures 2A and B, 3A and B). Diffuse ‘ground-glass’ opacity was seen bordering the thickened septae. Towards the periphery of the lungs, several smaller areas of pulmonary ‘honeycombing’ were visible (Figure 3A and B). These changes differed markedly from the reticular pattern described in a normal tortoise [12,13]. Incidental, multiple round soft tissue opacities up to 1.3 cm in diameter (mean HU of 70), surrounded by well-defined hypoattenuating rim (mean HU 20), were seen in the mid to caudal ventral half of the coelomic cavity. The CT findings were compatible with chronic, extensive ‘interstitial’ pulmonary fibrosis with follicular stasis.

Additionally, CT of another Leopard tortoise of approximately similar size and age was performed for comparison (Figures 2C and D, 3C and D). This tortoise was euthanized due to a traumatic left humeral fracture and the lungs were confirmed to be normal on histopathology. The normal reticular pattern of the lungs was appreciable on CT.

## Outcome and histopathologic findings

**Figure 4:**
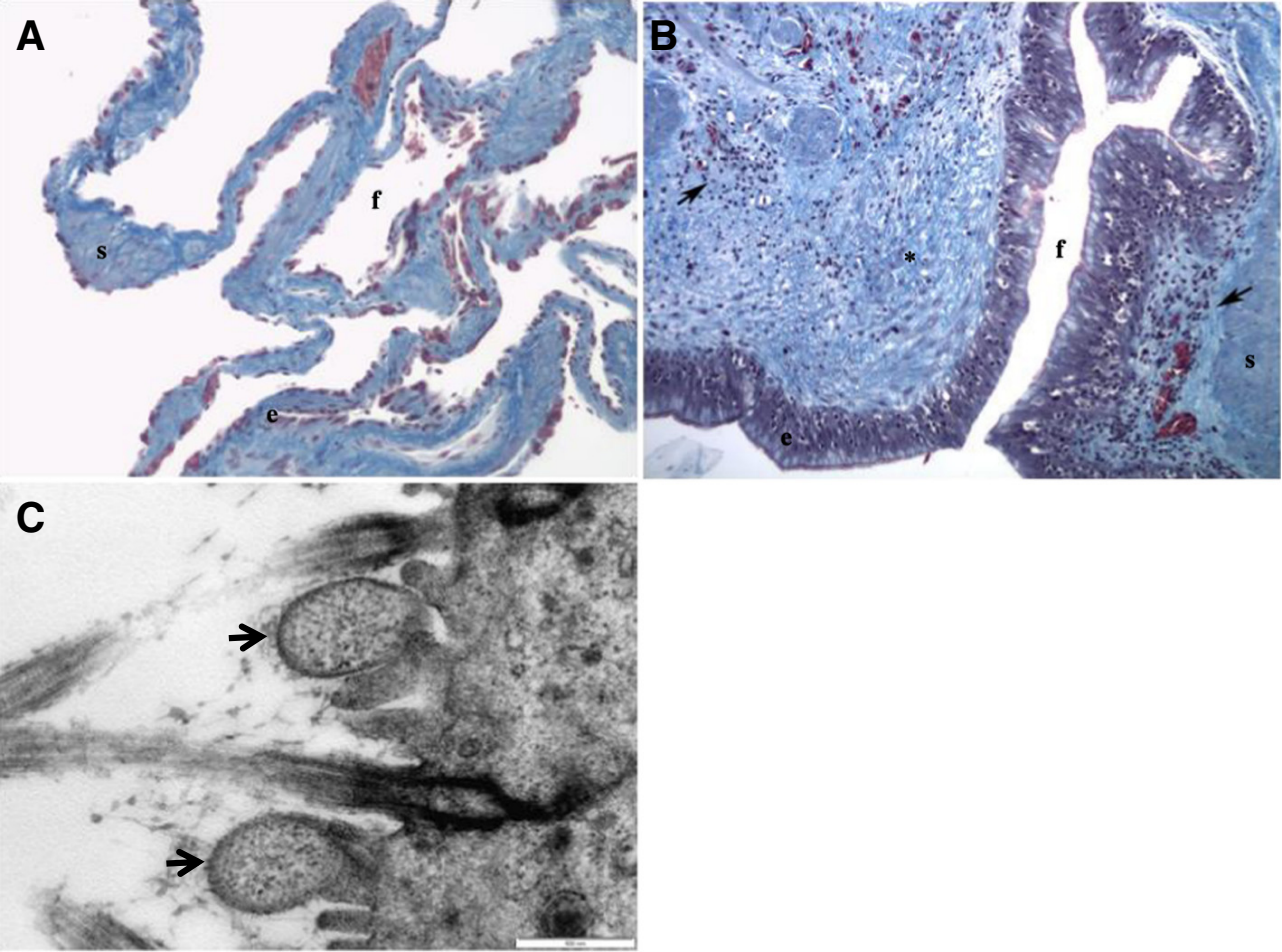
**Histological section of a normal lung of another Leopard tortoise compared to histological section of the lung of the affected Leopard tortoise with Mycoplasma organism seen under electron microscopy.** (**A**) Normal lung, (**B**) chronic hyperplastic pneumonia in a Leopard tortoise and (**C**) Mycoplasma organism (arrows) seen under electron microscopy. Note the hyperplastic epithelium (**e**), presence of inflammation (arrows) and fibrosis (*) in the affected tortoise. Faveolar spaces (**f**) and smooth muscle (**s**) are included for reference. Masson’s trichrome, ×10.

The patient was euthanized due to the extensive and severe pulmonary changes. Formalin fixed lung samples obtained at necropsy were examined histologically, after routine processing and staining, and compared to the normal lungs (Figure 4A) obtained from the euthanized Leopard tortoise with a fractured humerus. Marked epithelial hyperplasia characterized by tall pseudostratified ciliated epithelium lining the faveolar septae was noted. Moderate mucous hyperplasia was present as evidenced by prominent basophilic blebs on the luminal surface of the epithelium. Septae were markedly thickened due to congested blood vessels and infiltrated by moderate numbers of lymphocytes, plasma cells, heterophils, macrophages laden with lipofuschin and small number of lymphoid follicles. Masson’s trichrome staining showed moderate to severe fibrosis characterized by increased amounts of collagen around septal blood vessels and forming the matrix of the thickened septae (Figure 4B). Electron microscopy revealed the presence of Mycoplasma organisms among the microvilli and cilia of the respiratory epithelium (Figure 4C). No protozoa, bacteria or Mycobacteria were seen in the tissues with special stains. A single 1 mm diameter fungal necrogranuloma was also present. The histopathologic diagnosis was chronic hyperplastic pneumonia.

## Discussion

The leopard tortoise (*Geochelone pardalis pardalis)* is included in the class Reptilia and the order of Chelonia or Testudines [17]. The chelonian respiratory tract is divided into upper and lower respiratory tracts [18]. Inspired air enters the upper respiratory tract through the nares with the mouth closed. The lower respiratory tract is made up of the glottis, larynx, a short trachea, paired bronchi and paired multichambered lungs. Both lungs occupy the dorsal coelomic cavity in which the dorsal surface is adhered to the peritoneal lining of the dorsal coelomic cavity. Ventrally, the lungs are separated from the coelomic viscera by a non-muscular *septum horizontale* or ‘pseudodiaphragm’, to which they are attached. The paired bronchi enter the dorsal aspect of the lungs where they branch repeatedly to terminate into an open air space where the spongy faveolar tissues open into [18,19]. Respiratory tract disease in chelonians is often multifactorial and coupled with environmental inadequacies. Many start off as subclinical disease with typical clinical signs such as open-mouth breathing, wheezing, dyspnea and nasal discharge occurring only in the advanced stages. Concomitant systemic signs may include lethargy, anorexia and weight loss.

Inciting causes of respiratory diseases in chelonians include infectious agents such as viruses (e.g. *Herpes virus*), bacteria (e.g. *Mycoplasma agassizii* and *Pasteurella testudines*)*,* fungi (e.g. *Candida albicans, Aspergillus* and *Penicillium spp*) and parasites (e.g. Coccidiosis and Spirorchis) while non-infectious causes will include direct trauma (e.g. crushing injuries) and neoplasia (e.g. pulmonary fibromas) [18].

The major problem when treating chelonians with respiratory disease is the late recognition of illness by the owner thus requiring additional diagnostic tests to fully elucidate the extent of the respiratory disease.

Although radiography is useful in imaging the dyspneic chelonian, provision of a definitive diagnosis can be challenging due to superimposition of anatomical structures [12,13]. Computed tomography is practically feasible in chelonians as chemical restraint is not required and CT accurately demonstrates the internal anatomy without superimposition of adjacent structures [12]. The slow respiratory rate also limits motion artifacts, thus allowing more accurate definition of the typical reticular lung pattern and pathologic findings [13,14,20].

The radiographic findings of an unstructured ‘interstitial lung pattern’ and CT findings of ‘ground-glass’ opacity, ‘honeycombing’ and thickened pulmonary septae in this case are similar to those described in canine idiopathic pulmonary fibrosis and the histopathologic findings were also similar [5,6,10]. In humans with idiopathic pulmonary fibrosis, the ‘ground-glass’ opacity is attributable to active inflammation of the alveolar walls or presence of fibrosis [21]. The ‘ground-glass’ opacity in this case corresponded to the prominent and thickened faveolar septae due to marked epithelial hyperplasia, inflammation and fibrosis.

The chronic ‘interstitial’ pneumonia may have been caused by Mycoplasma infection. Chronic conjunctivitis and rhinitis due to Mycoplasma infection has been reported in various captive and free-ranging tortoises and turtles in the United States and Europe [22]. However, lower respiratory tract lesions due to Mycoplasma infection and the presence of these organisms in the lower respiratory tract have not been previously described. No other pathogens could be found on histology or electron microscopy, but resolving bacterial or viral pneumonia, helminth migration, thermal injury, inhalation of irritant gases, or ingested toxins could not be ruled out. Additionally, in humans, the possible role of immune complexes derived from non-specific antigens attracting polymorphonuclear leukocytes and macrophages by means of chemotactic process have been implicated in the etiopathogenesis of idiopathic pulmonary fibrosis [23]. The single fungal granuloma found in this case was most likely secondary to compromised mucosal integrity. Chelonians have been shown to be very poor at clearing secretions and foreign material from their lower respiratory tracts [19]. This is largely due to the fact that they are unable to elicit a cough reflex as they lack a muscular diaphragm. In most animals, respiratory mycoplasmosis tends to be a slowly progressing, chronic and seemingly clinically silent condition until it is exacerbated by environmental factors, stress or other microbial agents [24,25].

The principal therapy for idiopathic pulmonary fibrosis is corticosteroids [26]. Anecdotal use of antifibrotic and immunosuppressive agents such as colchicines, penicillamine and cyclosporine has been reported but not thoroughly evaluated in human and veterinary medicine [26,27]. Nevertheless, the efficacy of the above mentioned therapy against idiopathic pulmonary fibrosis is arguable and only provides symptomatic relief at best. Pirfenidone, an antifibrotic drug with anti-inflammatory properties has been approved by the European Commision in 2011 for treatment of pulmonary fibrosis in humans [3] but the use of this drug for pulmonary fibrosis is not validated in veterinary medicine.

## Conclusions

This report documented the first case of radiographic and CT findings of chelonian pulmonary fibrosis with histopathologic confirmation. This is also the first time Mycoplasma organisms have been found in the lower respiratory tract of a chelonian. The reported findings may assist in the antemortem diagnosis of pulmonary fibrosis for other chelonians.

## Consent

Written informed consent was obtained from the owners for publication of this report and any accompanying images.

## Abbreviations

CT: Computed tomography; WL: Window level; WW: Window width; MPR: Multiplanar reformatted; HU: Hounsfield unit.

## Competing interests

The authors declare that they have no competing interests.

## Authors’ contributions

CKL carried out the diagnostic imaging procedures and interpretation and is the main author of the paper. RMK made an intellectual contribution and reviewed the paper. EPL performed the histopathologic examination and interpretation. DLE is the referring clinician for the case. All authors read and approved the final manuscript.

## References

[B1] Heikkilä HP Lappalainen AK Day MJ Clercx C Rajamäki MM Clinical, bronchoscopic, histopathologic, diagnostic imaging, and arterial oxygenation findings in West Highland white terriers with idiopathic pulmonary fibrosis J Vet Intern Med 2011 25 433 439 10.1111/j.1939-1676.2011.0694.x 21366693

[B2] Heikkilä HP Krafft E Jespers P McEntee K Rajamäki MM Clercx C Procollagen type III amino terminal propeptide concentrations in dogs with idiopathic pulmonary fibrosis compared with chronic bronchitis and eosinophilic bronchopneumopathy Vet J 2013 196 52 56 10.1016/j.tvjl.2012.07.023 22909956

[B3] Cottin V Changing the idiopathic pulmonary fibrosis treatment approach and improving patient outcomes Eur Respir Rev 2012 21 161 167 10.1183/09059180.00001112 22654089 PMC9487299

[B4] Nalysnyk L Cid-Ruzafa J Rotella P Esser D Incidence and prevalence of idiopathic pulmonary fibrosis: review of the literature Eur Respir Rev 2012 21 355 361 10.1183/09059180.00002512 23204124 PMC9487229

[B5] Corcoran BM Cobb M Martin MWS Dukes-McEwan J French A Luis Fuentes V Boswood A Rhind S Chronic pulmonary disease in West Highland white terriers Vet Rec 1999 144 611 616 10.1136/vr.144.22.611 10390801

[B6] Lobetti RG Milner R Lane E Chronic idiopathic pulmonary fibrosis in five dogs J Am Anim Hosp Assoc 2001 37 119 127 11300517 10.5326/15473317-37-2-119

[B7] Cohn LA Norris CR Hawkins EC Dye JA Johnson CA Williams KJ Identification and characterization of an idiopathic pulmonary fibrosis-like condition in cats J Vet Intern Med 2004 18 632 641 15515577 10.1892/0891-6640(2004)18<632:iacoai>2.0.co;2

[B8] Secrest SA Bailey MQ Williams KJ Smarick SD Imaging diagnosis-feline idiopathic pulmonary fibrosis Vet Radiol Ultrasound 2008 49 47 50 10.1111/j.1740-8261.2007.00316.x 18251294

[B9] Krafft E Heikkilä HP Jespers P Peeters D Day MJ Rajamäki MM McEntee K Clercx C Serum and bronchoalveolar lavage fluid endothelin-1 concentrations as diagnostic biomarkers of canine idiopathic pulmonary fibrosis J Vet Intern Med 2011 25 990 996 10.1111/j.1939-1676.2011.0766.x 21781169

[B10] Johnson VS Corcoran BM Wotton PR Schwarz T Sullivan M Thoracic high resolution computed tomographic findings in dogs with canine idiopathic pulmonary fibrosis J Small Anim Pract 2005 46 381 388 10.1111/j.1748-5827.2005.tb00334.x 16119057

[B11] Lynch DA Godwin JD Safrin S Starko KM Hormel P Brown KK Raghu G King TE Jr Bradford WZ Schwartz DA Richard Webb W High-resolution computed tomography in idiopathic pulmonary fibrosis: diagnosis and prognosis Am J Respir Crit Care Med 2005 172 488 493 10.1164/rccm.200412-1756OC 15894598

[B12] Gumpenberger M Henninger W The use of computed tomography in avian and reptile medicine Semin Avian Exot Pet Med 2001 10 174 180 10.1053/saep.2001.24674

[B13] Gumpenberger M Computed tomography (CT) in chelonians Proceedings of the Association of Reptilian and Amphibian Veterinarians (ARAV) 9th Annual Conference, Reno, Nevada, 9–12 Oct 2002 2002 41 43

[B14] Gumpenberger M Schwarz T, Saunders J, West S Chelonians Veterinary computed tomography 2011 UK: Wiley-Blackwell 533 544

[B15] Saber AS Kamal BM Computed tomography and 3D reconstruction of the respiratory organs of the Egyptian tortoise (*Testudo kleinmanni*) J Vet Anat 2010 3 1 15

[B16] Valente ALS Cuenca R Zamora M Parga ML Lavin S Alegre F Marco I Computed tomography of the vertebral column and coelomic structures in the normal loggerhead sea turtle (*Caretta caretta*) Vet J 2007 174 362 370 10.1016/j.tvjl.2006.08.018 17084649

[B17] Keymer IF Diseases of chelonians: (1) Necropsy survey of tortoises Vet Rec 1978 103 548 552 10.1136/vr.103.25.548 105454

[B18] Origgi FC Jacobson ER Diseases of the respiratory tract of chelonians Vet Clin North Am Exot Anim Pract 2000 3 537 549 11228895 10.1016/s1094-9194(17)30088-9

[B19] Murray MJ Mader DR Pneumonia and respiratory function Reptile medicine and surgery 1996 Philadelphia: WM Saunders 396 406

[B20] Gumpenberger M Seybold J, Chimaira MF Diagnostic imaging of dyspnoic chelonians Proceedings of the 7th International Symposium on Pathology and Medicine in Reptiles and Amphibians (Berlin 2004) 2007 217 222

[B21] Leung AN Miller RR Muller NL Parenchymal opacification in chronic infiltrative lung diseases: CT-pathologic correlation Radiology 1993 188 209 214 8511299 10.1148/radiology.188.1.8511299

[B22] Jacobson ER Jacobson ER Bacterial disease of reptiles Infectious diseases and pathology of reptiles – Color atlas and text 2007 Boca Raton, FL: CRC/Taylor and Francis Group 461 526

[B23] Corcoran BM Idiopathic pulmonary fibrosis: an emerging disease in dogs Congress synopses: 22nd World Small Animal Veterinary Association Congress, Birmingham, UK, 2–4 Apr 1997 1997 42

[B24] Simecka JW Davis JK Davidson MK Ross SE Stadtlander CTKH Cassell GH Maniloff J, McElhaney RN, Finch LR, Baseman JB Mycoplasmas which infect animals Mycoplasmas: molecular biology and pathogenesis 1992 Washington DC: American Society for Microbiology 391 415

[B25] Weinach OM Snoeyenbos GH Smeyser CF Soerjude-Liem AS Influence of *Mycoplasma gallisepticum*, infectious bronchitis, and cyclohexamide on chickens protected by native intestinal microflora against *Salmonella typhimurium* or *Escherichia coli* Avian Dis 1985 28 416 425 6331365

[B26] Reynolds HY Isselbacher E, Braunwald JD, Wilson JB, Martin AS, Fauer DL, Kasper KJ Interstitial lung diseases Harrisons’s principles of internal medicine 1994 13 New York: McGraw-Hill 598 607

[B27] Du Bois RM Idiopathic pulmonary fibrosis Annu Rev Med 1993 44 441 450 10.1146/annurev.me.44.020193.002301 8476263

